# Nanopharmaceuticals for Eye Administration: Sterilization, Depyrogenation and Clinical Applications

**DOI:** 10.3390/biology9100336

**Published:** 2020-10-14

**Authors:** Aleksandra Zielińska, Beatriz B. Soles, Ana R. Lopes, Beatriz F. Vaz, Camila M. Rodrigues, Thais F. R. Alves, Dorota Klensporf-Pawlik, Alessandra Durazzo, Massimo Lucarini, Patricia Severino, Antonello Santini, Marco V. Chaud, Eliana B. Souto

**Affiliations:** 1 Department of Pharmaceutical Technology, Faculty of Pharmacy, University of Coimbra, Pólo das Ciências da Saúde, Azinhaga de Santa Comba, 3000-548 Coimbra, Portugal; zielinska-aleksandra@wp.pl (A.Z.); bbarreto17@hotmail.com (B.B.S.); anarosavieiralopes@gmail.com (A.R.L.); beatriz-vaz@hotmail.com (B.F.V.); camilamrodrigues8@gmail.com (C.M.R.); 2 Institute of Human Genetics, Polish Academy of Sciences, Strzeszyńska 32, 60-479 Poznań, Poland; 3 Laboratory of Biomaterial and Nanotechnology (LaBNUS). University of Sorocaba, Raposo Tavares 92.5, Sorocaba, 18078-005 São Paulo, Brazil; thaisfrancinealves1@gmail.com; 4 Institute of Quality Science, Poznań University of Economics and Business, al. Niepodległości 10, 61-875 Poznań, Poland; dorota.klensporf-pawlik@ue.poznan.pl; 5 CREA-Research Centre for Food and Nutrition, Via Ardeatina 546, 00178 Rome, Italy; alessandra.durazzo@crea.gov.it (A.D.); massimo.lucarini@crea.gov.it (M.L.); 6 Center for Biomedical Engineering, Department of Medicine, Brigham and Women& Hospital, Harvard Medical School, 65 Landsdowne Street, Cambridge, MA 02139, USA; pattypharma@gmail.com; 7 Biotechnological Postgraduate Program, University of Tiradentes (Unit), Av. Murilo Dantas, 300, 49010-390 Aracaju, Brazil; 8 Institute of Technology and Research (ITP), Nanomedicine and Nanotechnology Laboratory (LNMed), Av. Murilo Dantas, 300, 49010-390 Aracaju, Brazil; 9 Tiradentes Institute, 150 Mt Vernon St, Dorchester, MA 02125, USA; 10 Department of Pharmacy, University of Napoli Federico II, 80131 Napoli, Italy; 11 CEB—Centre of Biological Engineering, University of Minho, Campus de Gualtar, 4710-057 Braga, Portugal

**Keywords:** eye administration, ocular drug delivery, nanopharmaceuticals, ophthalmic treatments, sterilization, depyrogenation

## Abstract

**Simple Summary:**

Nanopharmaceuticals have revolutionized the way ophthalmic drugs are administered to overcome ocular delivery barriers and improve drug bioavailability. The design and production of an efficient ocular drug delivery system still remain a challenge. In this review, we discuss the sterilization and depyrogenation methods, commonly used for ophthalmic nanopharmaceuticals, and their clinical applications.

**Abstract:**

As an immune-privileged target organ, the eyes have important superficial and internal barriers, protecting them from physical and chemical damage from exogenous and/or endogenous origins that would cause injury to visual acuity or even vision loss. These anatomic, physiological and histologic barriers are thus a challenge for drug access and entry into the eye. Novel therapeutic concepts are highly desirable for eye treatment. The design of an efficient ocular drug delivery system still remains a challenge. Although nanotechnology may offer the ability to detect and treat eye diseases, successful treatment approaches are still in demand. The growing interest in nanopharmaceuticals offers the opportunity to improve ophthalmic treatments. Besides their size, which needs to be critically monitored, nanopharmaceuticals for ophthalmic applications have to be produced under sterilized conditions. In this work, we have revised the different sterilization and depyrogenation methods for ophthalmic nanopharmaceuticals with their merits and drawbacks. The paper also describes clinical sterilization of drugs and the outcomes of inappropriate practices, while recent applications of nanopharmaceuticals for ocular drug delivery are also addressed.

## 1. Introduction

**Figure 1:**
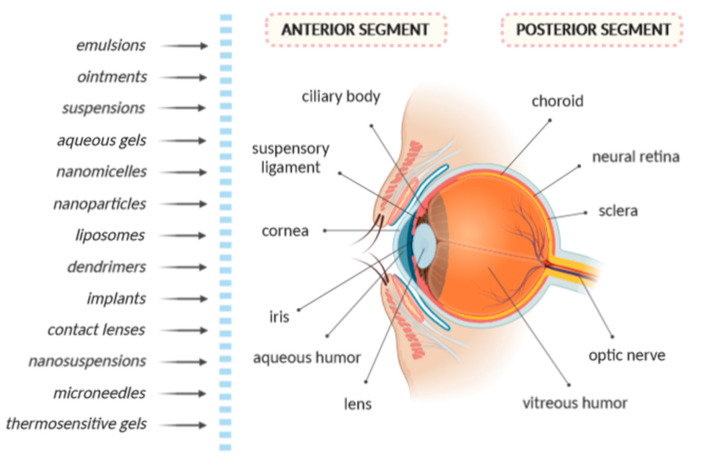
Structure of the human eye and various form of drug delivery systems.

The structure of the eye can be split into two main parts, namely, the anterior segment and the posterior segment [1,2]. As shown in Figure 1, the anterior segment includes the iris, ciliary body, cornea, conjunctiva, aqueous humor and lens, whereas the choroid sclera, neural retina, retinal pigment epithelium, vitreous humor and optic nerve belong to the posterior segment. Visual complications and blindness are usually related to several diseases of the posterior segment of the eye, in particular, in the retina.

Due to the complex anatomy and physiology of the eye, the delivery of therapeutic agents into its inner structures represents a great challenge. It is commonly accepted that an ideal delivery system should ensure effective drug levels over the timeframe of the treatment upon a single application [2]. The eye offers multiple routes through which ocular drugs can be delivered. Drug delivery to the anterior segment of the eye is currently achieved throughout topical and subconjunctival routes or injected intracamerally [2]. A topical eye drop is the most convenient and patient compliant route of drug administration, especially for the treatment of anterior segment diseases [3,4]. However, the ocular bioavailability is less than 5% of the topically applied dose [3,4].

The use of topical eye drops to achieve therapeutic drug concentration into the posterior segment thus represents a great challenge; several other pathways of drug administration, including periocular injections, intravitreal injections and systemic administration, are usually employed. Bearing in mind the fibrous composition and large surface area, the sclera offers less resistance to drug diffusion. Kang-Mieler’s group has shown that molecules up to 70 kDa can readily penetrate the sclera, whereas only those below 1 kDa penetrate through the cornea [2].

As they have limited adhesion onto the ocular surface, topical eye drops often show insufficient drug retention; besides that, little is known about the effect of efflux transporters on the corneal surface. Several approaches to improve corneal resistance to nasolacrimal drainage have been explored, such as the use of viscosity builders and corneal penetration enhancers by inhibiting p-glycoprotein. If, on the one hand, the effect of viscosity in liquid preparations is limited due to the clearance rate, on the other hand, the overly sensitive nature of the corneal and conjunctival tissues requires imposing caution to prevent toxicity [5,6].

Different conventional and innovative drug delivery systems, such as ointments, emulsions, aqueous gels, suspensions, nanoparticles, nanomicelles, dendrimers, liposomes, implants, contact lenses, nanosuspensions, microneedles and in situ thermosensitive gels, have been developed with the aim to overcome the ocular barriers and improve drug bioavailability in eye tissues [7]. Advances in nanotechnology have resulted in the development of ocular formulations based on biodegradable microparticles and nanoparticles, hydrogels and also implants, resulting the improvement of the bioavailability of several ocular drugs [2].

The knowledge of a patient’s hygienic habits is particularly important for clinical ophthalmologists, since pathogens have been identified even in the tears of asymptomatic patients [8]. Because of the intimate contact with the eye tissues, not only the drug delivery systems but also the ophthalmic tools used in clinical practice should be free from microorganisms. Sterilization is thus mandatory for both drug delivery systems and ophthalmic tools. For instance, the use of the ocular tonometer (i.e., for the measurement of the intraocular pressure by eye care professionals) is associated with the most common ophthalmic nosocomial outbreaks [9,10]. The test involves the touching of an anesthetized cornea by a tonometer tip that aims to record the amount of force required to flatten the cornea. To implement sanitization of this instrument, major guidelines are required, differing on procedures to disinfect tonometers. The recommendations from the World Health Organization indicate the use of 3–6% hydrogen peroxide for cleaning tonometer tips. However, according to the Guidelines for Disinfection and Sterilization in Healthcare Facilities, 3% hydrogen peroxide and 70% isopropyl alcohol are ineffective against adenoviruses. The adoption of disposable tonometer tips, particularly for patients suspected of having prion diseases, is recommended by the scientific literature, since it has been proven that no tonometer disinfectant is fully efficient against prions [9]. 

Nanopharmaceuticals for eye administration, known as complex systems, which are made of multiple components, should necessarily be sterile [11,12,13]. Thus, the manufacturing process must be sterile or terminal sterilization should be included in the process, while the further testing of nanopharmaceuticals in clinical trials is related to the approval of the production methods and quality assurance of the final product [11,12,14]. Besides the physical methods, such as irradiation, filtration or autoclaving, there are also chemical treatments, including the use of hydrogen peroxide, gas plasma, ethylene oxide and chemical vapor [15,16]. All of these methods, however, may have a negative influence on the physical/chemical characteristics of nanopharmaceutical dosage forms.

The choice of the sterilization technique is instrumental to ensure antimicrobial safety and the extended shelf life of the product. Furthermore, well-optimized sterilization parameters highly protect the product against degradation and can limit the final amount of toxic residues released.

Preformulation studies designed for the development of sterilized nanopharmaceuticals also need to ensure that the process can be scaled up [16,17]. Another challenge in producing nanopharmaceuticals is the fact that, apart from the sterilization, the product must be pyrogen free [17]. This is monitored by performing endotoxin assays on the drug products. Endotoxins trigger the immune system and activate the release of proinflammatory mediators which lead to endotoxin shock, tissue injury and sometimes death [18], outcomes that depend on the amount of endotoxin present [17]. Most nanopharmaceuticals interfere with the available endotoxin assays, which reduces the reliability of the test results. Therefore, other assays may be required. Non-sterilized conditions for nanopharmaceuticals can be beneficial, although it is important to start the production with sterile, pyrogen-free raw materials to ensure that production is carried out under microorganism-free conditions [16]. Since nanopharmaceuticals have revolutionized the way of producing new drug formulations and their administration, it is possible to overcome the ocular drug delivery barriers and improve ocular bioavailability of several drugs. In this review, we discuss the sterilization and depyrogenation methods, commonly used for ophthalmic nanopharmaceuticals, and their clinical applications.

## 2. Sterilization Methods of Ophthalmic Nanopharmaceuticals

In order to prevent disease transmission related to the use of the product, the sterilization that refers to the destruction of microorganisms (e.g., viruses, fungi, spores) is recommended. The process using ethylene oxide (ETO) at a low temperature has been the most commonly applied since the 1950s for sterilizing temperature/moisture-sensitive ophthalmic healthcare facilities in the United States. Moreover, this method has been validated by the sterilizer manufacturer for the specific instruments, in relation to potential ocular toxicity, efficacy of sterilization and instrument functionality [10].

**Figure 2:**
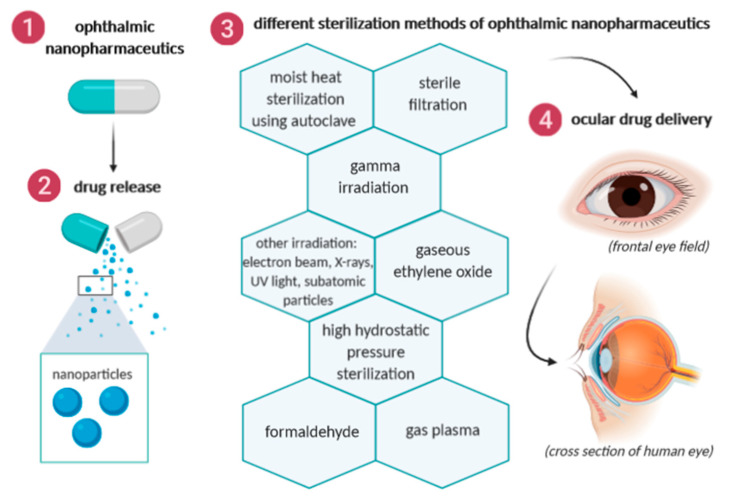
Different sterilization methods of ophthalmic nanopharmaceuticals.

Different sterilization methods of ophthalmic nanopharmaceuticals are illustrated in Figure 2 and they are further described in the sections below. Advantages and drawbacks of each method, as well as their most significant effects, are summarized in Table 1.

### 2.1. Moist Heat Sterilization Using Autoclave

An autoclave, based on moist heat sterilization, is a piece of equipment used for killing microorganisms [19]. Autoclaving, as an efficient method to inactivate bacteria, viruses and other biological material, is recommended for disposal regulated medical waste [20,21]. Depending on the high temperature (around 120 °C), chemical and structural changes in nanopharmaceuticals may occur. These modifications are mainly observed for nanopharmaceuticals that contain heat-sensitive drugs. Physical changes of nanopharmaceuticals, such size and morphology, can also be caused by their autoclaving, which can lead to variation of the amount of drug loaded in nanoparticles and the rate of drug release [16]. Both lipid-based and self-assembled nanoparticles, as well as those with a glass transition and melting point below 120 °C, are reported to be the most impacted by moist heat [16]. Both the increase in temperature during this process and the cooling will lead to rearrangement of nanoparticles’ molecular structure. Moreover, even in the presence of surfactants or stabilizers that reduce the risk of particles’ aggregation, the internal structure of the particle should not be affected by the nanopharmaceuticals’ sterilization method [16]. High temperatures used in autoclaving have a destructive influence on the nanoparticles based on matrix materials of low melting point. By reducing the temperature, the autoclaving time can be prolonged in order to counteract the degradation of the nanoparticle’s matrix induced by heat, ensuring the sterilization efficacy [16].

Vetten et al. (2014) [17] have summarized the challenges encountered in the sterilization of nanoparticles, for instance, the risk of aggregation, size increase of the nanoparticles and modifications on their surface charge. The authors demonstrated that all identified changes were dependent on the polymer or surfactant used in the production of particles, as well as on the production method. Besides the physicochemical changes, the moist heat sterilization in autoclaving can also lead to changes in biological effects [22,23,24].

If nanopharmaceuticals are sterilized using the autoclave, it is necessary to verify if the autoclaved product suffered any thermal degradation. Autoclaving is considered acceptable in the case of no changes in the nanoparticles. Therefore, the morphology of nanoparticles, their long-term stability, particle size distribution, loading capacity and the rate of drug release should be evaluated in parallel [16].

**Table 1:** Overview of ophthalmic nanopharmaceutical sterilization methods.

Sterilization Methods	Effect	Advantage	Drawbacks	References
Autoclaving: high pressure steam	bactericidal	low cost	chemical degradation, structural modification	[16]
Filtration: barrier	physical retention	drug thermally sensitive	viscosity, size	[17,25,26,27]
Gamma irradiation: ionizing	damage of genetic material	viscous material, drug and adjuvant thermally sensitive, no residue, effective against bacteria, yeast, fungus	chemical degradation, free radical, rate of drug delivery, gas formation, high cost	[16,28]
Gaseous ethylene oxide	bactericidal	low cost, drug and adjuvant thermally sensitive	toxic residue, cascade of oxidation, chemical change	[29]
High hydrostatic pressure	affect the cellular structures or functions	bar-resistant nanoparticles (polymeric carriers)	modifies adsorption, physical and chemical stability	[30]
Formaldehyde	bactericidal, but highly toxic	low cost	toxicity (truncates proteins) and carcinogens, affect the re-dispersion	[17,31,32]
Gas plasma: oxide reduction effect	antimicrobial	low temperature, non-toxic	oxidative, aggregation	[15,17,30]

### 2.2. Sterile Filtration

Sterile filtration is one of the most commonly used methods for the sterilization of nanoparticles, especially those that are heat labile and within a small particle size range [33]. However, the viscosity of the solution limits the nanopharmaceuticals that can be sterilized by filtration. Due to their small size, nanoparticles are able to penetrate through 0.22 µm filters that may lead to distortion of the particles and/or loss of the loaded drugs. A selective retention of the active ingredient on the fiber is another inconvenience that can occur [16]. In turn, physically removing microorganisms from thermally and chemically sensitive liquids is commonly applied using 0.22 µm membrane filters, thereby helping to protect the structure of nanoparticles [17]. Notwithstanding, this method cannot be used if the particles are larger/similar to the pore size of the filters since clogging may occur, resulting in a decreased yield [25,26]. In order to overcome this problem, the size of the nanoparticles should be designed to be less than 0.15 µm. Nagai et al. (2014) have described the biopharmaceutical advantages of ophthalmic nanoparticles ranged between 60 and 80 nm [27]. When this sterile filtration method is used, nanopharmaceuticals must be evaluated for changes in particle size and distribution, drug loading, composition of the formulation and viscosity changes [16].

### 2.3. Gamma Irradiation 

Gamma irradiation is indicated for nanopharmaceuticals that are thermosensitive, and can be used in the sterilization of sealed packaged products. Gamma irradiation is a type of ionizing radiation of short wavelength that carries enough energy to free electrons from atoms or molecules. It causes damage to the DNA or RNA of bacteria, fungi, yeasts and viruses [34,35].

Gamma irradiation can induce the degradation of nanoparticles, and/or physicochemical modifications, such as crosslinking and chain scission in the matrix of nanoparticles. These changes can influence the rate of drug release from the nanoparticles. The main changes observed after gamma irradiation of polymers and lipids are related to the gas formation, double bond, busted radicals, color formation, crystallinity, thermal transition and change in the surface hydrophilicity—hydrophobicity [28]. One of the major problems is the production of reactive free radicals. In particular, if water occurs during the process, it can lead to a series of free radical reactions. These radicals may interact both with the matrix of nanoparticles and with the loaded drug, resulting in physicochemical changes of the structure of the drug and in its interaction with the nanoparticles’ matrix [16].

The irradiation induced by degradation is affected not only by the chemical structure of the polymer, but also by its physical form [36]. Moreover, it is necessary to consider not only the influence of the gamma radiation on the polymers composing the nanoparticles but also on their physicochemical stability. The drug may be sensitive to the gamma irradiation, even if the polymeric material is resistant. The formulation buffer can also affect the peroxide formation. For each type of nanopharmaceutical formulation, establishment of the gamma irradiation sensitivity is required [16]. Several approaches exist to reduce the effects of gamma irradiation, such as optimization of the irradiation dose, removal of water by freeze-drying, using gamma radiation-resistant excipients, including free radical scavengers in the formulation, removing the oxygen or applying a vacuum during irradiation [16]. Monitoring the level of free radicals is necessary, while the toxicity profile of the nanopharmaceuticals must be fully tested [16]. Besides the identified shortcomings, gamma irradiation is still a method of interest for the sterilization of nanopharmaceuticals, because the results (e.g., shelf-life of the product, toxicity profile and induced responses) are not significantly changed in most cases [16,34].

### 2.4. Other Irradiation Methods

Several other irradiation methods, such as electron beam, X-ray radiation and UV light have also been proposed for the sterilization of nanoparticles. They are independent from heat and chemicals and do not leave residue after sterilization [17]. For electron beam sterilization, very high electron energy is used for a shorter duration when compared to gamma irradiation [17]. It is very effective, although it can damage the material being sterilized (similar to gamma irradiation). Some of the disadvantages of this method include changes in size, morphology, molecular mass and the pH of nanoparticles, degradation of antimicrobial and anti-sedimentation agents and changes in release profile, however, these changes do not always adversely affect the loaded drug [31]. Moreover, UV light irradiation is indicated for sterilization of surfaces using a germicidal lamp [31]. This method is effective in preventing bacterial growth, but it can only be applied if the nanoparticles’ properties remain unchanged [17].

### 2.5. Gaseous Ethylene Oxide

Gaseous ethylene oxide is usually used for devices that are not heat tolerant. This method works by alkalizing proteins and nucleic acids. Among some disadvantages, chemical changes in the drug or in the nanoparticles’ matrix, as well as the interactions between them, may be mentioned. Harmful gas residues may remain on the surface or within the nanoparticles, causing hemolysis. Additionally, in living organisms, ethylene oxide produces covalent adducts with DNA that cause mutagenic and genotoxic effects [37]. Some treated materials can generate toxic residues or set off a cascade of free radical reactions in the nanoparticles’ matrix material, in the drug or both. Therefore, after being sterilized, nanopharmaceuticals must be tested for residues of the gas and other toxic degradants [38].

The ethylene oxide can also promote changes in the rate of drug release, long-term stability and in the toxicological profiles of nanopharmaceuticals [16]. Friess and Schlapp reported aggregation and changes in the release profile of drug-loaded nanoparticles when sterilized with ethylene oxide, thereby demonstrating that this method can destructively influence nanopharmaceuticals [39].

### 2.6. High Hydrostatic Pressure Sterilization

It has been proven that a high hydrostatic pressure sterilization was ineffective in the process of spore elimination in high-pressure-resistant nanoparticles [16]. A high hydrostatic pressure up to 500 MPA for 30 min induces neither physical nor chemical damages, except on the surface modifiers that can be absorbed onto the nanoparticles. Brigger et al. (2003) have proposed high hydrostatic pressure as an innovative methodology for the sterilization of drug polymeric carriers, also underlying how further exploitation in this field is needed to develop novel protocols for spore inactivation [30].

### 2.7. Formaldehyde

Formaldehyde is an organic solution commonly used as a disinfectant and as a fixative [17]. It can be useful for materials that are sensitive to high temperatures, however, it is toxic and carcinogenic [28]. Sommerfeld et al. (1998) reported that aseptic manufacturing would be preferential over formaldehyde for sterilization purposes [32].

The chemical sterilization of poly(butylcyanoacrylate) nanoparticles by formaldehyde was shown to be inappropriate because of the difficulty in re-dispersing the freeze-dried particles obtained with different stabilizers, after formaldehyde treatment at 60 °C [32].

### 2.8. Gas Plasma

Gas plasma can be defined as an ionized gas that has properties of both gases and liquids. It has been shown to have a broad spectrum of antimicrobial effects [17], can be used at low temperatures and it is non-toxic [40]. Although its mechanism of action is not fully understood, it is believed that gas plasma is related to oxidation and reduction effects on microbial structures [41]. Some adverse effects, e.g., aggregation and alteration of the coating, have been observed due to the high oxidative nature of gas plasma [15].

## 3. Endotoxin Contamination

Bacterial endotoxin or lipopolysaccharide (LPS) is produced by Gram-negative bacteria and is known to be a potent inflammatory mediator causing septic shock syndrome, it can cause diffuse lamellar keratitis and is the main factor for toxic anterior segment syndrome (TASS). Gram-negative bacteria (predominantly *Sphingomonas*, *Ralstonia* and *Pseudomonas*) can form biofilms on the surface of objects and in formulations. Although Gram-negative bacteria can be destroyed by short-cycle sterilization methods, endotoxins are released from their bacterial cell walls and can remain on the surface of objects (e.g., ophthalmic instruments) and in the formulations. Endotoxins are heat-resistant and can remain biologically active, posing serious concerns for the safe use of such tools, including nanopharmaceuticals. The traditional test used to quantify endotoxin contamination is limulus amebocyte lysate (LAL). It has been used as an official test to replace the rabbit pyrogen test. The LAL test has three formats: chromogenic, turbidity and gel clot [17]. Although the formal acceptance requirements for the validity of LAL are in conformity, it is possible to find different results for the same nanoparticles. As concluded by Dobrovolskaia et al. [42], when these differences in LAL results are higher than 35%, the outcomes are verified in vivo using the rabbit pyrogen test. This problem can occur due to nanoparticle interference with the reactivity of the endotoxin in the LAL reaction [17]. Moreover, as marked by Smulders et al. [43], it is also important to include controls to help recognize the cause of negative results.

The endotoxins cannot be removed by the traditional filtration method as they will easily pass through the membrane filter. Thus, other types of filtration are used in the pharmaceutical industry, such as chromatography and ultrafiltration, to remove them [18]. However, before using these methods, it is required to ensure that nanopharmaceuticals have no contaminants and do not interact with the columns [17]. Due to the complexity of nanoparticles and endotoxin, there is no efficient technique for their removal. The Food and Drug Administration (FDA) has recommended either applying high temperatures or high concentrations of acids/bases, but these conditions will likely affect the properties of nanopharmaceuticals. In this case, a contaminant-free production process is recommended [17].

Depyrogenation refers to the removal or inactivation of pyrogens. The depyrogenation method is chosen according to the procedure to be performed to remove endotoxin from nanopharmaceuticals [17,18,44,45,46,47]. Table 2 shows methods used for depyrogenation and its main features.

## 4. Sterilization of Ophthalmic Nanopharmaceuticals

**Figure 3:**
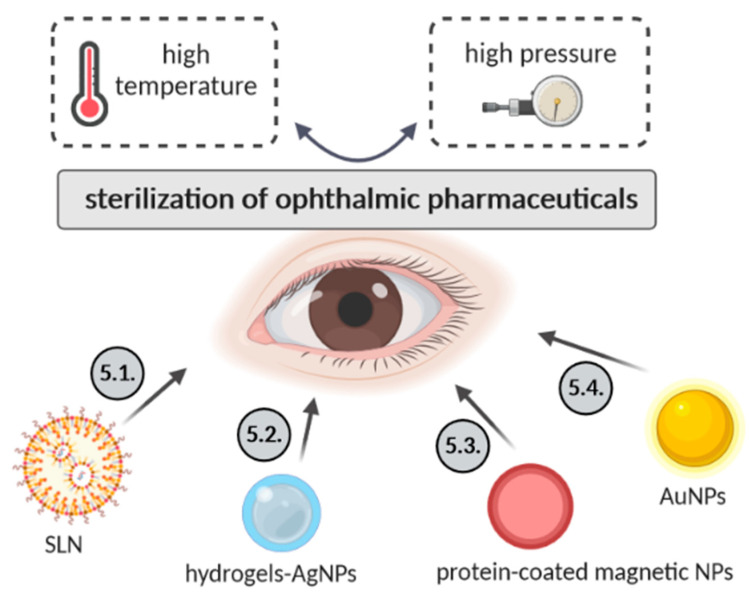
Different types of nanoparticles used as ophthalmic nanopharmaceuticals.

Several sterilizations methods of ophthalmic pharmaceuticals based on different types of nanoparticles (lipid-based, silver, magnetic or gold nanoparticles) are here described and summarized in Figure 3. 

### 4.1. Solid Lipid Nanoparticles

Solid lipid nanoparticles (SLNs) have been proposed as colloidal drug carriers for several administration routes [48,49,50,51], including eye administration [3,4,52,53,54,55,56]. They offer several advantages, such as bioacceptable and biodegradable composition, small size, no toxicity, high loading capacity or controlled drug delivery [54,55,56,57,58]. SLNs can be produced using several methods, among which high-pressure homogenization and the microemulsion method at high temperatures may act as sterilizing approaches [59,60]. Cavalli et al. (1997) [61] have developed SLNs by dispersing warm oil-in-water microemulsions in a cold aqueous medium under mechanical stirring [61]. Upon lipid recrystallization, a dispersion of SLNs is obtained from the oil droplets. To obtain dry products and provide longer stability, SLN dispersions should be lyophilized, although this process can also lead to changes in the mean size and shape of nanoparticles [61].

Several sterilization approaches have been used in lipid nanoparticles. The autoclaving of SLNs for 15 min at 121 °C under pressure of 2 bar has been carried out by Cavalli et al. according to the European Pharmacopoeia II, showing that SLNs were stable during the process and maintained a spherical shape and narrow size distribution [61]. Moreover, no particles larger than 1 µm were recorded in the samples stored at 4 °C for more than a year. The high temperatures reached during autoclaving presumably created a hot oil/water microemulsion, which was then recrystallized in the form of SLNs within the nanometer size range [61]. No differences were observed between a drug-loaded SLN compared with the drug-free SLN [61]. The average diameter (Z-Ave) and polydispersity index (PI) slightly increased, while the zeta potential (ZP) mostly remained the same [61].

To ensure a spherical shape without any significant increase in the size or particle size distribution, SLNs can be sterilized by autoclaving. Although the stability of the lipid matrix should not be affected, SLNs loaded with heat-resistant drugs should be sterilized by a transition temperature above that of the autoclaving [16].

### 4.2. Hydrogels Containing Silver Nanoparticles

Hydrogels (HGs) are described as three-dimensional and crosslinked polymer networks. They are able to retain large quantities of water [14]. Among their most important features, their high biocompatibility, possibility of modulating mechanical properties and production facilities, are highlighted [62].

Hydrogel-based ophthalmic formulations are widely reported to improve corneal drug retention time. One of the barriers for ophthalmic gel formulations is the sterilization of dosage forms without interfering with the drug content, microbiological and chemical stability. To overcome the gel sterilization, the use of in situ stimuli-responsive gelling in response to environmental change (trigger), such as temperature triggered, ionic concentration, enzymes or pH lacrimal, has been proposed. For this gelling system, the sterilization of the dosage form would be in a value below that of the low critical system temperature, based on conformational changes in the polymer structure by weak acid or basic groups that accept protons in responsive changes (pH triggered), and by click reaction for ionotropic crosslinking that takes place due to single or double replacement reactions [63,64].

Silver nanoparticles (AgNPs) are used as a reservoir of silver ions for antimicrobial applications [65,66,67,68]. They have higher antibacterial activity when compared to free ions, since AgNPs are protected against inactivation by biological fluids. The dispersion of AgNPs into HGs has been proposed as topical formulation with antimicrobial properties. These nanosystems are highly sensitive to the sterilization process, thus the method used should not alter mechanical, functional and chemical properties of either the polymers or encapsulated drug [62].

Rafael et al. (2019) [62] produced and characterized thermo-sensitive HGs based on Pluronic^®^ F127 loaded with AgNPs. Dry heat methods affected the quality of these nanoparticles but not the autoclaving methods. A direct sterilization of HG-AgNPs by autoclaving has been proven to be effective. The filtration technique was also tested as a terminal sterilization, but was not effective due to the viscosity of the polymer solution.

According to Rafael et al. (2019) [62], both the polymer and HG are more sensitive to sterilization by dry heat, resulting in the loss of their gelation properties. The autoclaving did not influence these properties, and it only led to a slight increase in the gelation time of the polymer [62]. The formation of reactive oxygen species reached higher values of peroxides when sterilized by dry heat than by autoclaving. The sterilization of the polymer prior to the HG formation led to the formation of peroxides [62]. The presence of AgNPs did not affect the gelation behavior when compared to the sterilized unloaded HG, but it has been proven that temperature can change the mechanical properties of the hydrogels. When temperature increased from 4–22 °C to 37 °C, morphology and microstructure changes could be noticed. Regarding the antimicrobial efficacy of sterilized vs. nonsterilized HGs and AgNPs, no significant differences were observed [62].

To sum up, HGs are mainly sensitive to conventional sterilization methods. Some of them cause physicochemical alterations that may impact on the mechanical properties and biocompatibility, as well as on the efficacy of the loaded therapeutic compounds [69]. Pre-processing of the polymers has affected mechanical properties of the HG. Furthermore, the dry heat sterilization has led to an increase in oxygen reactive species, which correlated with a reduction of the biocompatibility of the HG. The formation of oxygen reactive species was also decreased. Therefore, a terminal steam heat was chosen as the most beneficial method for HG sterilization, due to a successful scaling up in production. In addition, using terminal steam heat at an industrial level, it is possible to overcome the need of production under sterile conditions.

### 4.3. Serum Protein-Coated Magnetic Nanoparticles

Magnetic nanoparticles are commonly used for imaging analysis [70], and for this purpose they should also be sterilized. Thanks to a protein coating, interactions between nanoparticles and biological systems may occur [71]. Zhao et al. (2010) [72] described no changes in Z-Ave, PI or zeta potential under UV radiation exposure of titania nanotubes. The increasing exposure time may have led to a reduction of the protein amount on the particle surface. A decrease in particle size was not related to protein degradation [71], even if the compaction of the proteins could be induced by radiation [73,74]. No influence of UV exposure on protein amount on the surface of the magnetic nanoparticles was shown by the measurements of the physical properties [71].

Dutz et al. (2017) [71] have shown a decrease in the thickness of the protein corona during autoclaving, while before and after UV exposure, a similar degree of damage to proteins has been noticed. No relevant changes in protein content and integrity have been observed after UV exposure (at 200–280 nm) for up to 240 min for protein-coated magnetic-based NPs [71]. Coagulation of the proteins caused a higher density of the proteins and, thus, a lower volume [74]. The components of the protein corona can thus form a denser layer after autoclaving. This can be explained by the coagulation of the corona proteins, but also because of the denaturation and extensive degradation of the proteins [71].

Autoclaving is not suitable for the sterilization of protein-coated magnetic nanoparticles using a standard protocol. It has been proven that this method can damage the integrity of the protein coating and influence its stability [71]. No destructive effects for protein-coated magnetic nanoparticles were reported for sterilization by using UV radiation. Thus, UV sterilization followed by lyophilization with the addition of polyethylene glycol can be a promising procedure for the sterilized long-term durable protein-coated magnetic nanoparticles [71].

### 4.4. Gold Nanoparticles (AuNPs)

França et al. (2010) [15] have tested different sterilization techniques by studying the physicochemical properties of two types of AuNPs upon sterilization. Ethylene oxide has been shown to be the most appropriate, because none of the tested AuNP samples reacted with the organic compound [16].

It has been proven that sterilization of Au-tiopronin nanoparticles by gas plasma and ethylene oxide did not influence the morphology and particle size distribution, whereas nanoparticle aggregation and coalescence into larger, irregularly shaped particles were caused by UV and formaldehyde sterilization. On account of the growth and recrystallization processes induced by the high temperatures used during the treatment, autoclave sterilization was responsible for the increase in particle size [75]. Neither of these sterilization procedures led to the loss of organic weight fraction nor affected inter-cluster hydrogen bonding between adjacent tiopronin molecules. On the other hand, the sterilization of polyethylene glycol (PEG)-coated AuNPs (Au-PEG-NPs) with gas plasma led to a change in solution color due to particle aggregation. The coalescence phenomena into larger particles of irregular shape was also observed. Formaldehyde and autoclave sterilization procedures led to a small fraction of particle aggregation and minor coalescence. UV irradiation and ethylene oxide treatment did not alter the nanoparticles. The stability of the particles is provided by the PEG. During the analysis, changes, such as degradation of the PEG shell, could lead to nanoparticles’ aggregation or coalescence. Except for the case of gas plasma, the same characteristics after sterilization processes were noticed. Gas plasma is highly oxidative and has a direct influence on PEG chemical stability [76].

As for cytotoxicity and reactive oxygen species (ROS) induction, AuNPs have not shown any important alterations after sterilization. It has been concluded that Au-tiopronin-NPs were not affected and the biocompatibility remained the same. On the other hand, Au-PEG-NPs caused a slight decrease in cell viability, which has been noticed for Au-PEG-NPs sterilized by formaldehyde. This is possibly due to the changes in the particles’ stability, and the presence of formaldehyde residues in the nanoparticles’ suspension. It is worth underlining that other methods have shown no impact on the cell viability. Regarding the induction of ROS, results have shown that all sterilized nanoparticles induced very low levels of intracellular ROS. In the case of Au-tiopronin-NPs treated by gas plasma and ethylene oxide, insignificant levels of ROS were induced. As for Au-PEG-NPs, a small amount of intracellular ROS was induced during the exposition of UV irradiation, ethylene oxide formaldehyde and by autoclaving. It has been concluded that ethylene oxide and gas plasma can be the best sterilization methods for Au-tiopronin-NPs. Gas plasma is not recommended for Au-PEG-NPs, because it has an impact on the PEG coating. Sterilization methodologies using UV irradiation and ethylene oxide may represent the most suitable procedures for AuNPs [15].

## 5. Guidelines for Cleaning and Sterilization

Toxic anterior segment syndrome (TASS) is one of the major problems associated with the presence of bacterial endotoxins in instruments and products used in intraocular surgeries. It causes an inflammatory reaction, which may be visible within 12 to 48 h after surgery of a cataract/anterior segment [77]. Toxic endothelial cell destruction (TECD) syndrome is a variant of TASS. Even though TASS is rare, complications of cataract and other intraocular surgery can still occur [78]. The etiology of this syndrome may be multifactorial. Among potential causes of TASS, contamination of balanced salt solutions, osmotic concentration or ionic composition may be mentioned. Moreover, intraocular irrigating solutions with abnormal pH, denatured ophthalmic viscosurgical devices, intraocular antibiotics, metallic precipitates and topical ointments may also cause this intraocular inflammation [79]. The inadequate cleaning of intraocular surgery instruments, the use of enzymatic detergent in wrong concentrations, reutilization of products/instruments of single use and the lack of maintenance of autoclaves also pose a significant risk to patients [80].

One of the most important habits in the procedure of ophthalmic products is cleaning and decontamination, followed by disinfection or sterilization. In the final stage, packaging and sterilization may follow. Ophthalmic tools should be carefully and singly cleaned, without any contact with other equipment [78].

Enzymatic detergents commonly contain subtilisin or alpha amylase exotoxins and neither are denatured by autoclave sterilization, which is the main reason why this is considered a controversial execution for the decontamination of intraocular surgical instruments. This is because studies both in animals and humans have shown corneal endothelial toxicity associated with the use of these detergents, and some outbreaks of TASS also suggest the inappropriate use of enzymatic detergents [8]. It is extremely important, when enzyme detergent is used, to follow an appropriate dilution and disposal of cleaning solutions. The instruments should be minutely flushed to assure the total removal of the detergent.

Ultrasonic cleaning is able to remove all visible soil before placing instruments in the ultrasonic cleaner [7]. The use of high-frequency as well as high-intensity sound waves in a liquid was able to remove contaminants from surfaces. The use of this instrument requires, however, some necessary daily procedures such as cleaning, disinfecting, rinsing and drying [80].

## 6. Sterilization of Ophthalmic Formulations

Sterilization is mandatory in order to reduce the risk of infection, but it is imperative to preserve the drugs and material properties.

Several studies report that it is permissible to autoclave surgical solutions after they are prepared. These reports have not included any changes that appeared under heat in many solutions [81]. Chlorobutanol (trichloro-2-methyl-2-propanol) and phenylethyl alcohol are used as ophthalmic preservatives. In addition to its antimicrobial activity, chlorobutanol also has an anesthetic effect in the eye. However, this preservative hydrolyzes and decomposes at autoclaving temperatures. Ophthalmic solutions should be prepared and preserved according to whether they are to be used in surgical procedures in the clinic or office and at home.

The addition of an inert, non-irritating, bactericidal and fungicidal agent, additionally compatible with the drug, is the most recommended methodology in order to maintain sterility of ophthalmic solutions [81]. However, there is always some deterioration. To provide sterility after autoclaving for ophthalmic solutions, it is necessary to add quaternary ammonium chloride solution. Studies have reported how it can maintain the sterility of solutions used in surgical procedures. Nevertheless, chloride may cause irritation, as well as produce edema and desquamation. All ingredients used for the preparation of ophthalmic solutions should be chemically pure. One of the earliest reports has shown that a 1:10,000 solution was safer to use for the preservation of ophthalmic solutions than a 1:3500 solution, which induced conjunctival and corneal changes [81]. In order to protect the product inside, colored bottles are widely used in clinical laboratories. Thus, drops for patients with glaucoma should be prescribed in small amounts and placed in these bottles—then replaced at least monthly [81].

## 7. Stability of Ophthalmic Formulations

The stability of solutions is especially important. Within one month after being dispensed at pH 8.3, a 44% decomposition of 0.5 wt% atropine solution and an 89% decomposition of a 1 wt% homatropine solution were found. Deterioration was less than 20% when they were dispensed at pH 6.8. It has been proven that slightly acid solutions of ophthalmic drugs were more stable and effective [81].

An additional consideration in adjusting the reaction is the relation between the pH of the solution and absorption by the eye and the fact that solutions of pH 7.3 and 9.7 have been studied in terms of sensitivity of the human eye, which only caused irritation in 1% of cases, while pH of 5.8 and 11.4 caused irritation in 99% of cases. This study also suggests that the eye can tolerate a considerably greater deviation from physiological pH towards alkalinity than towards the acid range. Human tears were proven to have low buffering capacity at physiologic pH. Hogan et al. (1949) [81] have already shown that absorption of 1 wt% of atropine solution at pH 4.0 was three times more efficient than at pH 7.5. In addition, pH change from 4.0 to 7.5 decreased the absorption of 0.5 wt% pilocarpine by 50 wt%. The same authors [81] also reported that pH change from 3.2 to 8.7 for 1 wt% of cocaine lead to an increase in its absorption by sevenfold.

Diclofenac, as eye drops, is a non-steroidal, anti-inflammatory, well-tolerated drug that has a poor aqueous solubility as sodium salt. Sodium salt of diclofenac is used for making aqueous ophthalmic solutions, a topical drug normally prescribed for ocular irritation, postoperative ocular pain and photophobia. Diclofenac sodium showed a tendency to precipitate from aqueous solutions in a crystalline form even when the concentration was under the limit of saturation. As observed and reported by Ahuja et al. (2009) [82], the formation of inclusion complexes of diclofenac with β-cyclodextrin can improve the thermal stability of diclofenac sodium in aqueous solutions [82].

Studies have been conducted on aqueous ophthalmic solutions of diclofenac. Consequently, it has been shown that 0.1 wt% of diclofenac formulation with pH 7.4 contained sodium chloride (tonicity equivalent), could provide a shelf life (t90) of 2 years thanks to a special preservative. As a result, the formulations seem to be promising for corneal permeation. However, for use in cataract surgery, a formulation without preservative or a formulation containing 0.1 wt% of diclofenac sodium (pH 7.4) and sodium chloride would be the best option [82].

## 8. Conclusions

The chemical and physical properties of nanopharmaceuticals are influenced by the selected sterilization methods. Selected procedures impact on nanopharmaceuticals to a certain extent. Therefore, there is a need to underline the importance of testing different/various methods to establish the specific type of sterilization for each type of nanoparticle. The optimization of the selected method for each formulation based on an acceptable level of product degradation, drug loss and shelf life should be also considered. Filtration has the lowest impact on formulations and it is a good option if nanoparticles can be recovered from the filtrate, while UV irradiation does not affect the drug release, but affects the physicochemical properties of nanoparticles. Autoclaving results in the aggregation of nanoparticles, but formaldehyde and ethylene oxide, as well as gas plasma, do not have enough information available, therefore their use is not recommended. It has been shown that ethylene oxide may especially cause extensive nanoparticle degradation. There is no single process that can equally be applied to all nanoparticles. It is recommended to prevent contamination. Small changes in nanopharmaceuticals may result in different effects upon sterilization. The sterilization and depyrogenation methods commonly used in industrial practices have here been reviewed, discussing their impact on nanopharmaceuticals. The most appropriate procedure should ensure the sterility, but also maintain the stability and physicochemical characteristics, of the nanopharmaceuticals. Following sterilization, the toxicity and functionality of nanoparticles must always be tested to ensure that the nanoparticles’ properties have not been affected. It also should be underlined how an appropriate stability testing should be established for the detection of chemical degradants during the shelf life of nanopharmaceuticals.

## Figures and Tables

**Table 2:** Methods used to remove endotoxin (depyrogenation).

Methods	Principle of the Method
Ultrafiltration	It eliminates the endotoxin by molecular weight using ultra-fines (10,000 Daltons or greater). It shows relatively good endotoxin clearance.
Reverse osmosis	This process uses a filter and highly pressurized conditions. It captures 99.5% of endotoxin and ions or salts but allows water molecules through. It is commonly used to produce highly purified water.
Two-phase partitioning	In this system, an aqueous surfactant solution spontaneously separates into two predominantly aqueous, but immiscible, forms in an effective separation.
Affinity chromatography	This method acts to bind endotoxin through biding affinity using ligands such as DEAE Sepharose, poly-L-lysine and polymyxin-B. Moreover, this method can be affected by the pH range, temperature, flow rate and the number of electrolytes in the solution.
Distillation	Endotoxin is removed by the rapid evaporation of the water molecules and the persistence of the larger lipopolysaccharide molecules in the original environment.
Adsorption	The endotoxin molecule is attracted to the activated carbon bed. This mechanism is less efficient and is affected by several environmental factors.
Acid-base hydrolysis	Occurs in the binding of lipid A with the polysaccharide nucleus. The isolated molecule is insoluble in an aqueous medium. The main acids used are HCl and glacial acetic acid diluted.
Plasma discharge	The UV radiation used in this process is responsible for the inactivation of spores. The main advantage is the possibility of the operation of the process at moderate temperatures, allowing the treatment of heat-degradable materials.
Oxidation	Depyrogenation occurs by peroxidation of the fatty acid in the lipid A region (e.g., using hydrogen peroxide)
Ethylene oxide	The process is performed in a heated, pressurized chamber, but at a lower temperature. The depyrogenation process occurs by nucleophilic substitution in the glucosamine of lipid A.
Moist heat	Traditional autoclaving does not destroy endotoxins. However, when combined with hydrogen peroxide and pressure, it is effective.
Dry heat	Endotoxins are destroyed by exposure to high temperature.
